# PREMM: preterm early massage by the mother: protocol of a randomised controlled trial of massage therapy in very preterm infants

**DOI:** 10.1186/s12887-016-0678-7

**Published:** 2016-08-27

**Authors:** Melissa M. Lai, Giulia D’Acunto, Andrea Guzzetta, Roslyn N. Boyd, Stephen E. Rose, Jurgen Fripp, Simon Finnigan, Naoni Ngenda, Penny Love, Koa Whittingham, Kerstin Pannek, Robert S. Ware, Paul B. Colditz

**Affiliations:** 1 Perinatal Research Centre, School of Medicine, Royal Brisbane & Women’s Hospital, Brisbane, Qld Australia; 2 University of Queensland Centre for Clinical Research, Level 4, Bldg 71/918, Royal Brisbane & Women’s Hospital, Brisbane, Qld Australia; 3 Grantley Stable Neonatal Unit, Royal Brisbane & Women’s Hospital, Brisbane, Qld Australia; 4 Stella Maris Institute, The University of Pisa, Pisa, Italy; 5 Queensland Cerebral Palsy and Rehabilitation Research Centre, School of Medicine, Faculty of Medicine and Biomedical Sciences, The University of Queensland, Brisbane, Qld Australia; 6 CSIRO, Australian e-Health Research Centre, Royal Brisbane and Women’s Hospital, Brisbane, Qld Australia; 7 UQ Child Health Research Centre, School of Medicine, The University of Queensland, Brisbane, Qld Australia; 8 School of Population Health, The University of Queensland, Brisbane, Qld Australia

**Keywords:** Preterm, Infant massage, Neurodevelopment, Attachment

## Abstract

**Background:**

Preterm infants follow an altered neurodevelopmental trajectory compared to their term born peers as a result of the influence of early birth, and the altered environment. Infant massage in the preterm infant has shown positive effects on weight gain and reduced length of hospital stay. There is however, limited current evidence of improved neurodevelopment or improved attachment, maternal mood or anxiety. The aim of this study is to investigate the effects of infant massage performed by the mother in very preterm (VPT) infants. Effects on the infant will be assessed at the electrophysiological, neuroradiological and clinical levels.  Effects on maternal mood, anxiety and mother-infant attachment will also be measured.

**Methods/Design:**

A randomised controlled trial to investigate the effect of massage therapy in VPT infants. Sixty VPT infants, born at 28 to 32 weeks and 6 days gestational age, who are stable, off supplemental oxygen therapy and have normal cranial ultrasounds will be recruited and randomised to an intervention (infant massage) group or a control (standard care) group. Ten healthy term born infants will be recruited as a reference comparison group. The intervention group will receive standardised massage therapy administered by the mother from recruitment, until term equivalent age (TEA). The control group will receive care as usual (CAU). Infants and their mothers will be assessed at baseline, TEA, 12 months and 24 months corrected age (CA), with a battery of clinical, neuroimaging and electrophysiological measures, as well as structured questionnaires, psychoanalytic observations and neurodevelopmental assessments.

**Discussion:**

Optimising preterm infant neurodevelopment is a key aim of neonatal research, which could substantially improve long-term outcomes and reduce the socio-economic impact of VPT birth. This study has the potential to give insights into the mother-baby relationship and any positive effects of infant massage on neurodevelopment. An early intervention such as massage that is relatively easy to administer and could alter the trajectory of preterm infant brain development, holds potential to improve neurodevelopmental outcomes in this vulnerable population.

**Trial registration:**

Australian New Zealand Clinical Trials Registry: ACTRN12612000335897. Date registered: 22/3/2012.

## Background

Over the past 50 years, there has been a progressive decrease in preterm infant mortality [1]. A lowering of the age limit of viability and an increase in preterm birth numbers has made preterm birth a significant public health issue [1]. Despite advances in technology, the number of preterm infants with neurodevelopmental compromise remains high [2]. In recent years the focus of improving preterm outcomes has shifted from increasing survival to minimising morbidity and improving neurodevelopmental outcomes [1, 3]. Long term neurodevelopmental abnormalities impact up to 50 % of these infants [4] and include motor disability (including cerebral palsy), reduced cognitive performance and behavioural problems [3]. The severity of the deficits is related to the degree of prematurity and the presence of neuroradiological injuries [3], however high rates of more subtle but nonetheless important neurodevelopmental abnormalities also occur in low-risk preterms without obvious brain injury [5, 6]. Even in late preterm infants, the effects of preterm birth on brain development are more significant and long lasting than previously thought [5, 7]. The untimely interruption of the developing fetus’ environment is thought to be a major contributor to this phenomenon [6].

### Disruption of optimal fetal environment

The altered neurodevelopmental trajectory observed in preterm infants results from interruption of the intrauterine environment. The neuromaturation of the cerebral cortex, initially laid down in cortical layers prior to 20 weeks gestation, is a dynamic process from 30 to 40 weeks gestation. It is during this time, the subplate, a transient population of neurons, guides the development of cortical and thalamocortical connections [8], maturing from its maximal prominence to almost complete regression. These connections are fundamental for cortical processing and cognition [8]. Between 29 and 41 weeks, total brain volume is increased nearly 3-fold, cortical gray matter volume is increased 4-fold and cerebellar volume is increased 4-fold [7]. Although some differences between term infants and preterm infants at term equivalent age are explained by the effect of complications associated with preterm birth, both the experience of a highly stressful ex-utero environment and the lack of the stimulation normally experienced in the womb, exert detrimental effects on the immature brain [6, 9]. The challenges imposed by the extra-uterine environment, disrupt the above described development of a “neurotypical” brain. Results include numerous primary medical problems experienced by preterm infants such as lung disease of prematurity, physiological instability, asphyxia, suboptimal nutrition, infection, medication side effects and hyperbilirubinaemia, each of which may have its own potentially deleterious impact on brain development [6, 10]. Environmental stressors, which include frequent noxious stimulation, excessive sound and constant light, also adversely affect the normal neurodevelopmental trajectory [6, 10]. Repetitive pain universally experienced by premature infants from frequent invasive procedures is hypothesised to cause excessive activation of central afferent pain pathways and excitotoxic damage to the developing brain [11]. While the impact of these factors and negative influence on the brain are recognised, emergent strategies to lessen harm include environmental enrichment and infant massage.

### Environmental enrichment

Environmental enrichment and social stimulation induce experience-dependent neuroplasticity in experimental animal models [10]. Neuroplasticity is the capacity of the mammalian brain to use the activity induced by a given experience, enabling the modification of function in neuronal circuitry [12]. Early interventions based on the manipulation of the extra-uterine environment, among them infant massage therapy, have been used in preterm infants with the aim of optimising the infant’s sensory experience and improving overall functional outcome [13, 14].

### Infant massage

Infant massage has been investigated as a potentially effective intervention aimed at providing a form of environmental enrichment [15]. It is often combined with other forms of stimulation such as kinaesthetic stimulation (e.g. passive extension/flexion movements of the arms and legs), talking or eye contact [16]. The possibility that infant massage could provide a form of comforting touch with positive effects on growth and neurodevelopment is described in a number of studies [16, 17]. Evidence supporting positive effects of massage in preterm infants include increased weight gain [18–20], improved growth and gastrointestinal function [21, 22], improved body fat deposition [23], improved neurobehavioural outcomes [17, 24–26], pain attenuation [27, 28], reduction of infant stress and stress-related factors [29, 30], reduction of late-onset sepsis [31], improved immune system [32], reduced jaundice [33] and improved heart rate variability [34] as well as a reduction in maternal depression and anxiety [35]. Studies investigating the use of specific oils in massage versus no oil suggest improved weight gain [36–38]. Overall the evidence remains weak, mainly due to small sample sizes, heterogeneity and poor methodology in some studies. The current level of evidence does not support wider use of infant massage without further research [17, 39]. Two key aspects of infant massage as an early intervention have received little attention. The first aspect is the effect of infant massage on direct measures of brain development, such as the maturation of brain electrical activity, brain structure and the relationship to clinical neurobehaviour. A recent meta-analysis reported that the association between massage and neurobehavioural development remained elusive [40]. The second aspect is the potential to enhance the efficacy of the intervention by active involvement of the parents; in particular the mother, and what effect of actively massaging her baby may have on the mother-infant relationship.

### Clinical neurodevelopmental assessments

Traditionally, methods of neurodevelopmental assessment of the preterm infant have included a number of clinical examinations used to evaluate neurological function and neurobehaviour [41]. Those most commonly used in the neonatal period include the Prechtl’s General Movements Assessment (GMA), Hammersmith Neonatal Neurological Examination (HNNE), Amiel-Tison Neurological Assessment at term, Neonatal Behavioural Assessment Scale (NBAS), Neurobehavioural Assessment of the Preterm Infant (NAPI) and the Neonatal intensive care unit Network Neurobehavioural Scale (NNNS) [42]. These assessments vary in the time required for training, as well as in the appropriate age of administration and scoring systems. This affects the ability to use them in clinical practice or research [42]. The Prechtl’s GMA has the greatest predictive accuracy for the diagnosis of cerebral palsy [41, 43] with a sensitivity of 98 % and specificity of 91 % [44]. The assessment is based on a global visual perception system of the quality and complexity of movements [45]. As such, it is an applicable tool to use evaluating the impact of massage.

Much like the rest of the brain, considerable development of the visual system occurs in the third trimester, which increases the potential for long-term visual dysfunction in preterm infants. The Neonatal Vision Scale is an assessment that was originally developed to test vision in full term infants. It has subsequently been applied to preterm infant cohorts to reliably measure the integrity of the visual system [46, 47]. Using this scale could assist in evaluating any measureable effects of massage on the visual system.

Measuring long-term neurodevelopmental outcomes has been standardised for a number of assessments for use in toddlers and young children. These assessments are particularly useful for assessing high-risk populations such as VPT infants. The Bayley Scales of Infant and Toddler Development, Third Edition (Bayley-III) is a structured instrument to assess cognitive and social-emotional development, language and motor abilities [48] and is the most widely used measure to assess neurodevelopment of VPT and very low birth weight infants in the first three years [49]. In a recent review, Mental Development Index (MDI) scores were strongly predictive of later cognitive functioning, r = 0.61 (95 % CI = 0.57-0.64) and motor scale scores were moderately predictive of later motor function, r = 0.34 (95 % CI = 0.26-0.42) [49]. In another recent study [25], 73 very low birthweight preterm infants who had been randomised to receive massage therapy or not, were followed up at 2 years of age with the Bayley Scales of Infant Development Second edition (BSID-II). Outcomes showed the Mental Development Index (MDI) was higher in the intervention group than the control group indicating better cognitive scores in the group who received massage [25]. In the present study, we will use Bayley-III to review these findings.

### Magnetic Resonance Imaging to assess structure

Magnetic Resonance Imaging (MRI) has become an essential neurodiagnostic tool as it offers high resolution images and can assist prognostication following neonatal brain injury [50]. MRI studies in preterm infants at term-equivalent age have shown that preterm birth alters development of regional brain volume [51], white matter [52–54], cortex [55, 56], deep gray matter [57, 58] and vascular organisation [59]. With diffusion tensor imaging, white matter integrity and white matter maturation can be studied, and white matter pathways can be non-invasively delineated through diffusion tractography [60–62]. Numerous studies have evaluated the ability of MRI at term equivalent age to predict neurodevelopmental outcomes at 1 to 9 years and established it as the best imaging tool available for outcome prediction of children born preterm [63]. To our knowledge, MRI studies of infants who have received massage therapy are yet to be described.

### Electroencephalography to assess function

Electrophysiological studies have reported significant differences in spectral electroencephalography (EEG) measures between healthy term and preterm infants [64–66]. Maturation of the EEG in preterm infants is characterised by decreases in the total amplitude and delta activity power in quiet and active sleep [67]. A preliminary study examining whether preterm infant massage has a beneficial effect on cerebral function as measured by EEG, found a significant difference in the interburst interval duration and also a difference in maturation of visual function in preterm infants who received massage in comparison to preterm infants who received standard care [68]. A subsequent study showed a relative increase in global EEG spectral power in delta and beta frequencies in massaged infants when compared to controls, which was interpreted as suggesting that massage therapy in low-risk preterm infants favors a process of maturation of brain electrical activity similar to that observed in term born infants [24].

### Parental stress, infant attachment and related assessments

The stress and trauma of VPT birth on the parents is well described [69–72]. Preterm delivery has been identified as a risk factor for stress, postpartum posttraumatic stress disorder, postpartum depression and difficulty with initial bonding and attachment [73]. The increased levels of stress, anxiety and depression may negatively influence the already difficult maternal-infant bonding [73]. Historically, a high dependence on technology for life-support, the institutionalisation of preterm infant care and the fear of infection have often resulted in the separation of mother and baby [74]. In the early 1970s, research demonstrated that mothers who were permitted to enter the nursery showed a greater commitment to their infants, were more confident in their mothering abilities and had increased caretaking skills [74]. In response to this and subsequent evidence, parental involvement was encouraged [74].

An increase in bonding and attachment behaviours and a decrease in parental depression has been reported in studies where mothers attended massage classes 2 months after birth at term [75]. In the same way, actively providing infant massage could help to mitigate the impact of stress and anxiety in the intensive care nursery environment, by allowing mothers to be more proactive in the developmental care of their babies. A variety of maternal factors including frequency of maternal touch and the degree of postpartum depression (PPD) can influence the neurodevelopmental and cognitive skills of the preterm infant [76, 77].

Maternal mood and anxiety have been measured using a number of scales. The most widely researched is the Edinburgh Postnatal Depression Scale [78] which demonstrates moderate to good internal consistency over several studies. Other measures such as the Postpartum Depression Screening Scale (PDSS) and the Beck Depression Inventory (BDI) are also widely used and demonstrate good concurrent validity [78].

The Depression Anxiety Stress Scale (DASS) is another widely studied assessment tool in the postnatal period. It is a 42-item questionnaire, completed by the mother, designed to measure the magnitude of these three negative emotional states [79, 80]. The Depression scale focuses on reports of low mood, motivation and self-esteem, the Anxiety scale assesses physiological arousal, perceived panic and fear, and the Stress scale measures tension and irritability [79, 80]. Internal consistency for each of the subscales is typically high (e.g. Cronbach’s α of 0.96-0.97, 0.84-0.92, 0.90-0.95 for Depression, Anxiety and Stress respectively) [80] and convergent and discriminant validity has been demonstrated [81].

Attachment difficulties can arise from preterm birth disrupting normal physical contact between the mother and infant [82] and withdrawal of the mother from their infant due to distress [83]. This may inhibit the mother’s caregiving behaviour as her desire and ability to provide protection for her preterm infant is interrupted, ultimately impacting the mother’s attachment representations and the child’s attachment patterns [84]. Infant Observation is a method developed by Esther Bick at the Tavistock Clinic in London more than 60 years ago, and has been used to understand the characteristics of the developing relationship between mother and infant [85]. This method has been widely used internationally for training and research in the psychodynamic-psychoanalytic field to capture the rich and complex nature of mother-infant attachment. One of these complexities in particular is that of maternal responsiveness.

Maternal responsiveness plays an important role in developing secure attachment and effective bonding patterns between mother and infant. Ineffective maternal responsiveness is directly related to a lack of attachment, low self-esteem and unhealthy growth and development of the child [82]. The Maternal Infant Responsiveness Instrument (MIRI) was designed to measure this concept by appraising the maternal awareness and reflection of the mother’s responsiveness to her infant and her recognition of her infant’s responsiveness to her. It is easy to administer and has an alpha reliability coefficient of 0.86 [82]. The Maternal Postnatal Attachment Scale (MPAS) is designed to evaluate maternal emotions and cognitions relating to attachment. It is specifically designed for use during the first year of life and focuses on the mother’s subjective experience in relation to her infant. Internal consistency as measured by Cronbach’s coefficient alpha is 0.78. In addition to maternal attachment, this research is also aimed to explore infant mental health.

Evaluating the infant’s social and emotional functioning can be an indirect measure of the integrity of infant attachment. It has been observed that secure infant attachment status is related to lower risk for later peer interaction and behavioral problems [86]. The Infant Toddler Social and Emotional Assessment (ITSEA) is a robust parent-report questionnaire designed to assess a wide array of social-emotional and behavioural problems and competencies [87]. Its psychometric properties are sound with strong test-retest reliability (0.61-0.91, mean = 0.79), with concurrent and discriminant validities (α = 0.69-0.86, mean = 0.76) [87].

In this project we will investigate several key aspects of the effect of infant massage on preterm neurodevelopment, its neurobiological correlates and the mother-infant relationship.

### Aim

The broad aim is to investigate the potential effects of infant massage performed by the mother, in VPT infants born between 28 and 32 weeks and 6 days gestational age. Effects on the infant will be assessed at the electrophysiological, neuroradiological and clinical levels at term equivalent age and 24 months corrected age. In addition, the impact of infant massage implemented by the mother on maternal mood and anxiety and infant attachment will be measured at term equivalent age, 12 months corrected and 24 months corrected age.

### Hypothesis

The primary hypothesis to be tested is that infant massage by the mother in VPT infants promotes favourable processes in brain development, which can be functionally measured with dense array electroencephalography (dEEG).

Secondary hypotheses to be tested include the following:

1. Infant massage by the mother in VPT infants promotes favourable processes in brain development, which can be detected structurally at term equivalent age, and functionally at term equivalent age and 24 months corrected age.
2. Infant massage by the mother in VPT infants reduces maternal depression, anxiety and stress associated with preterm birth.
3. Infant massage by the mother in VPT infants enhances infant attachment.

## Methods

### Design

A randomised controlled trial will be conducted to investigate the effect of infant massage. Infants will be recruited and randomised to an intervention group (PREMM), which will receive infant massage by the mother or a control group, which will receive care as usual (CAU). A study coordinator, who is not involved in generating the randomisation sequence, will assess infants for eligibility and obtain written informed consent from the parent(s).

### Participants

#### Preterm participants

Preterm infants born between 28 weeks and 32 weeks and 6 days gestational age, admitted to the Grantley Stable Neonatal Unit at the Royal Brisbane & Women’s Hospital will be assessed for eligibility. Parents will be approached for consent if a head ultrasound around day 10 after birth confirms the absence of moderate or severe intraventricular haemorrhage (> grade II) [88], periventricular echogenicity or periventricular cysts. Once written informed consent is obtained, baseline measures will be collected before allocation occurs. Allocation will be performed by opening the next, in sequence, opaque envelope by non-study personnel. In this way infants will be randomised into a massage group or a care as usual group.

##### Inclusion criteria

Infants born between 28 and 32 weeks and 6 days gestational age, with a birthweight between the 10^th^ and 90^th^ percentile (for their gestational age and gender), who are clinically stable, off oxygen therapy or respiratory support and have no IVH greater than grade II. The cut-off age for recruitment is 34 weeks and 3 days old, to ensure ample time for infants in the massage group to receive the intervention.

##### Exclusion criteria

The presence of abnormalities on brain ultrasound including intraventricular haemorrhage (grade III and IV), periventricular echogenicity or periventricular cysts. Infants with major genetic disorders and malformations will also be excluded.

### Sample size

Preliminary data from a pilot study, which described global spectral EEG power-associated brain maturation in massaged preterm infants, was used to calculate a sample size [24]. To find a 10 % between-group difference in EEG power in the treatment group [24], with type 1 error rate 0.05, and power of 80 %, we require 20 participants in each group to complete the study. Assuming that two thirds of participants will remain in the study at term equivalent age, we will require a total of 60 preterm infants to be recruited (i.e. 30 per group).

### Randomisation

Randomisation will be performed in Statistics Package for the Social Sciences (SPSS) 15.0 (SPSS Inc., Chicago, IL, USA) using random number generation with stratification by gender. The ratio of intervention to care as usual participants will be 1:1. A third party not involved with recruitment will generate the randomisation list and the list will be concealed. After random allocation lists have been generated, the allocation group (intervention or care as usual) of the premature infants will be stored in sequentially numbered, sealed, opaque envelopes. These envelopes will be opened by non-study personnel at each study enrolment, after baseline measures have been obtained.

### Intervention

The intervention will be introduced to the mother following allocation into the massage group. The massage paradigm is modified from the preterm massage protocol of Field et al. [89] that consists of 2 parts, a tactile phase and a kinaesthetic phase. Mothers will be taught the techniques and encouraged to massage their babies for 15 min, twice a day until term equivalent age. Those allocated to the control group will receive care as usual.

**Fig. 1:**
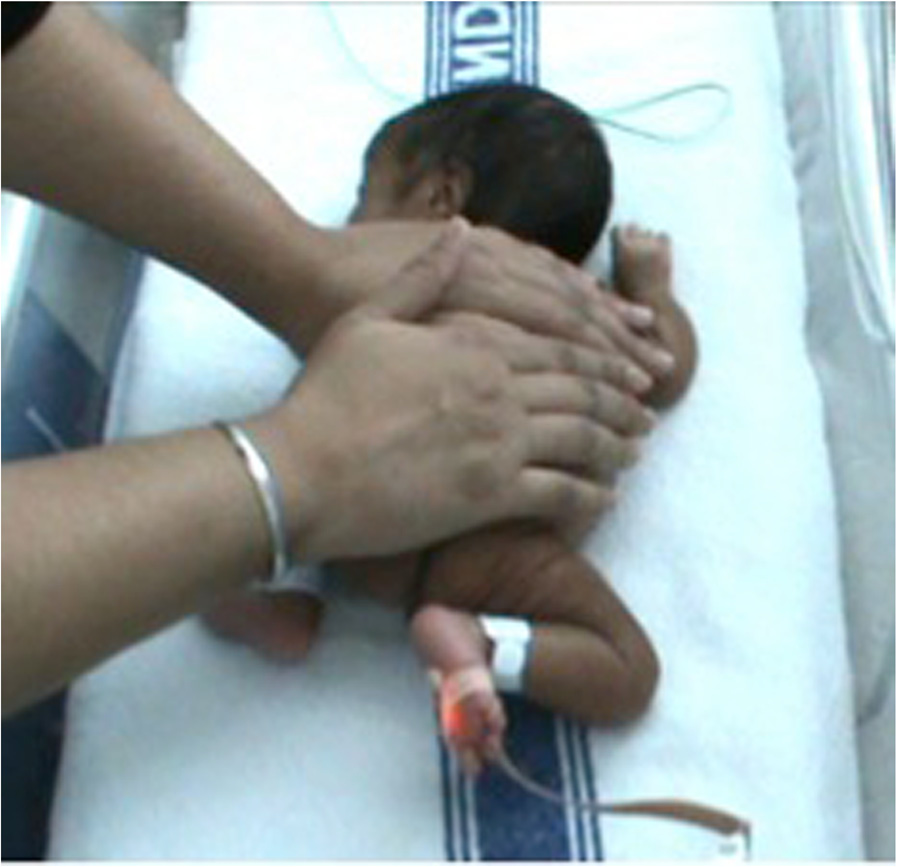
"Resting hands" prone position in massage protocol

Mothers will be given the following instructions both in demonstration and in written form for later reference. The tactile phase will begin with asking “baby’s permission”, in the prone position and enclosing the infant with cupped hands in a “resting hands” position (Fig. 1). A small amount of massage oil (cold-pressed fruit or vegetable oil) will be used. Maintaining a “resting hand” to the back, the mother will slowly stroke the baby with the palm of her other hand with a gentle but firm, rhythmic motion, following this sequence: head to the neck (6 times), shoulder to the hand (6 times) left and right, neck to the back to the bottom (6 times), bottom to the feet (6 times) left and right. After returning to “resting hands” position for up to a minute, this sequence will be repeated. During the massage the mother is to breathe deeply and slowly, keeping herself relaxed whilst paying attention to the baby’s stress cues which include crying, skin mottling, hiccups, gagging, apnoea and bradycardia. If signs of stress are evident, the mother will to return to “resting hands” until the baby settles and is ready for further massage. If the baby doesn’t settle, the mother will stop and re-attempt the massage later.

For the kinaesthetic phase, the baby is placed in the supine position. Again, the mother will place her palms on the baby’s body to embrace the chest in a “resting hands” position. If the baby’s eyes are open, eye contact and verbal interaction will be encouraged. Kinaesthetic stimulation will follow this sequence: 6 flexions and extensions of the right upper limb, 6 flexions and extensions of the left upper limb, 6 flexions and extensions of the right and left upper limbs together, 6 flexions and extensions of the right lower limb, 6 flexions and extensions of the left lower limb, 6 flexions and extensions of the right and left lower limbs together. After returning to “resting hands” position for up to a minute, this concludes the session. The massage sequence will be taught to the mother by the same paediatric physiotherapist trained in the Field method of massage [89].

The mothers will be provided with a structured diary to keep a record of the number and duration of each session. Massage sessions will be preferably performed around 60 min before feeding and at least 2 h after the completion of the previous session. Up to three individual teaching sessions will be given to each mother and support will be continued until the mother is considered to be competent and confident. Regular contact with the mother will ensure that she continues to use the outlined protocol. When the baby is discharged home, the mother will be asked to continue daily massages until TEA.

Infants of the CAU group will undergo routine nursery care and there will be no specific interventions. Intervention and CAU mothers will be assigned to different nursery rooms to prevent contamination. In addition, nursery staff will be briefed on the importance of maintaining the study protocol. Very close monitoring of the data collection process will ensure protocol violations will be identified and action will be undertaken to prevent this, in a sensitive manner.

#### Term participants

A term born reference group will be recruited from the postnatal wards or via interested parents for comparison to a normal infant population.

The term born reference group will be born between 37 and 41 weeks gestation following an uncomplicated pregnancy and delivery, have a birthweight > 10^th^ centile and have not required special care admission.

### Measures

#### Baseline measures

1. Demographics: Medical risk factors for outcome using standardised Australian and New Zealand Neonatal Network (ANZNN) data definitions [90] (e.g. gestational age, birthweight) will be collected from the baby’s case notes as baseline demographics.
2. Prechtl’s Qualitative Assessment of General Movement [45]: Video of the baby’s general movements will be collected for general movements assessment performed at a later date by a certified assessor who will be blind to group allocation.
3. Hammersmith Neonatal Neurological Examination (HNNE): The HNNE is a discriminative and predictive scale for the neurological examination of the preterm and term newborn [91–93]. It is derived from a combination and adaptation of items taken from Prechtl, Saint Anne Dargassies and Brazelton methods in a simplified and easy to administer protocol [92]. When compared to term born infants, preterm infants at term equivalent age have lower scores when assessed with HNNE [92]. The examination will be performed by a Paediatric Neurologist, Paediatric Physiotherapist or Neonatologist who will score the infant at the same time.
4. Depression Anxiety Stress Scales (DASS): This self-report measure consists of three 14-item scales assessing depression, anxiety and stress over the past week. Each item has 4 response options ranging from 0 (“Did not apply to me at all”) to 3 (“Applied to me most of the time”) [94]. If questionnaire scores indicate significant emotional stress, psychosocial support will be offered. Mothers will be asked to complete this assessment.
5. Edinburgh Postnatal Depression Scale (EPDS): The EPDS is a 10-item self-administered questionnaire developed for screening postpartum depression in women in outpatient or home visiting settings [95]. Psychosocial support will be offered and appropriate referrals made if the EPDS score is greater than 9. Mothers will be asked to complete this assessment.
6. Mother-to-Infant Bonding Scale (MIBS): The MIBS is designed for use from day one postpartum, offering the mother one-word descriptors of possible emotions towards her new child. It is quick and easy to use and has reasonable reliability (α score 0.71) [96]. Mothers will be asked to complete this assessment.
7. Infant Observations: These are conducted on mother-infant pairs, each of 1 h duration by one Psychoanalytic Psychotherapist trained in the Esther Bick Tavistock model of observation [85]. Each observation will be assessed using the following criteria: amount of touching by the mother, amount of visual checking if the mother is away from the infant or if the infant is asleep, amount of auditory checking, amount of talking about the infant by the mother, the infant's desire for contact with the mother, visual/physical reaching, anxiety apparent in the relationship, reports of illness or difficulties, willingness to let the observer see the infant. This will be the first of four such observations to evaluate the evolving mother-infant relationship.

### Preterm infants timing of assessments

All preterm infants will be asked to return to hospital for assessment at term equivalent age (39 to 42 weeks post menstrual age (PMA)), then further assessments at 12 and 24 months corrected age either at home or at the hospital (Table 1).

**Table 1:** Outcome measures for preterm infants

Measures for Preterm infants	Baseline	Term Equivalent Age	12mth corrected	24mth corrected
Clinical	Demographics HNNE GMA	HNNE GMA NVS		Bayley-III
Infant Observation	IO	IO	IO	IO
Questionnaires	DASS MIBS EPDS	DASS MIBS EPDS	MIRI MPAS	DASS ITSEA MSES
Radiological		MRI		
Electrophysiological		dEEG		

HNNE = Hammersmith Neonatal Neurological Examination; GMA = General Movements Assessment; NVS = Neonatal Vision Scale; Bayley-III = Bayley Scales of Infant and Toddler Development, 3^rd^ Ed; IO = Infant Observation; DASS = Depression, Anxiety, Stress Scale; MIBS = Mother to Infant Bonding Scale; EPDS = Edinburgh Postnatal Depression Scale; MIRI = Maternal Infant Responsiveness Instrument; MPAS = Maternal Postnatal Attachment Scale; ITSEA = Infant Toddler Social and Emotional Assessment; MSES = Maternal Self Efficacy Scale; MRI = Magnetic Resonance Imaging; dEEG = dense array electroencephalography

### Outcome measures

#### Primary outcome

The primary outcome measure is beta-frequency global power (EEG parameter) in preterm infants, at term equivalent age, who have received massage therapy or care as usual.

High-density electroencephalography (dEEG) will be recorded using a 64-electrode sensor net (HydroCel Geodesic Sensor Net, Electrical Geodesics Inc.). Each electrode is a saline sponge, in a geodesic tension structure comprised of silastic threads. The sensor net is prepared by soaking in a normal saline solution and then applied directly onto the head without need for further scalp preparation. The infant will be fed, wrapped and placed in an open cot, in a darkened room fitted with a Faraday cage. The recording will be conducted during sleep. EEG signals are amplified by a GES 300 series amplifier (Electrical Geodesics Inc.), digitised (at a sampling rate of 256 Hz) and recorded to the hard drive of a Mac desktop computer via NetStation software (Electrical Geodesics Inc.). The EEG data files will be exported from NetStation software then pre-processed and analysed quantitatively using Curry Scan 7 Neuroimaging Suite signal processing software (Compumedics Neuroscan™, Compumedics Limited, Australia).

### Secondary outcomes

i. Other EEG parameters at term equivalent age (TEA)
  Global power in delta, theta and alpha bands, absolute and relative power for each frequency band, interhemispheric coherence and partial directed coherence will be analysed.
ii. MRI measures at TEA
  A brain MRI will be performed using a 3.0-T (Siemens TIM Trio, Erlangen, Germany) and an MRI-compatible incubator with a dedicated head coil (LMT Lammers Medical Technology, Lubeck, Germany). The infants will be fed, fitted with earmuffs to minimise noise exposure (Natus Mini Muffs, Natus Medical Inc., San Carlos, CA) and monitored by pulse oximetry, then wrapped and placed in an MRI-compatible incubator to keep the infant still, warm and supported in the scanner. Scanning will occur for about 45 to 60 min without sedation.
  The MRI protocol will include T1 turbo spin echo (TSE), T1w MPRage, T2w HASTE and 3 echo T2 map, 30 direction diffusion weighted imaging (DWI), and 64 direction DWI sequences. A neuroradiologist will review clinical sequences and classify any white and grey matter [58, 97] abnormalities. Diffusion images will be acquired using single-shot echo planar multi-direction diffusion-weighted sequence, employing dual bipolar diffusion gradient and double spin echo. This will include the acquisition of a 30 direction DWI protocol (b = 1000s/mm^2^) and a 64 direction HARDI protocol (b = 2000s /mm^2^), in which the encoding gradients are uniformly distributed in space (acquisition time 5 min and 11 min respectively). A field map for diffusion data is acquired using two 2D gradient recalled echo images to assist in correction for residual distortions due to susceptibility in homogeneities (acquisition time 1 min). An extensive pre-processing and quality control procedure will be used to detect and correct image artefacts caused by head movement, cardiac pulsation, and image distortions [60]. Fractional anisotropy (FA) and mean diffusivity (MD) will be estimated from corrected diffusion data using a diffusion tensor model. Constrained spherical deconvolution implemented in MRtrix will be employed to estimate fibre orientation distribution (FOD) [98]. Whole-brain voxel based analysis of FA and MD will be performed using tract-based spatial statistics optimised for neonates [99]. Whole-brain voxel-based analysis of fibre orientation distributions will be conducted using Apparent Fibre Density (AFD) [100]. Probabilistic tractography will be performed using MRtrix. White matter pathways will be delineated using the multi-regions-of-interest approach. A number of pathways, including corticospinal tract, corpus callosum, superior longitudinal fasciculus and thalamic radiations will be extracted. Summary measures of FA, MD, AFD and T2 within pathways will be calculated and related to EEG and clinical findings.
iii. Preterm infant clinical measures
  8. Clinical assessments performed at baseline will also be performed at TEA; Prechtl’s Qualitative General Movements Assessment (GMA) [45] and Hammersmith Neonatal Neurological Examination (HNNE) [92]. All examinations will be performed by a Paediatric Neurologist, Paediatric Physiotherapist or Neonatologist blinded to allocation groups.
  1. A visual assessment will be performed at TEA. The Neonatal Vision Scale (NVS) [47] is a clinical assessment consisting of a short test battery that explores various aspects of visual function, ranging from the ability to fix and follow a target, to more complex aspects of visual function, such as reaction to a colour target, discrimination of black and white stripes with increasing spatial frequency and attention at a distance [47].
  2. The Bayley Scales of Infant and Toddler Development, Third Edition (Bayley-III) will be performed [48] at 24 months corrected age. Children will be assessed in the 5 key developmental domains of cognition, language, social-emotional, motor and adaptive behavior [48].
  3. The Infant Toddler Social and Emotional Assessment (ITSEA) will be measured at 24 months corrected age. The ITSEA is a 136-item parent report questionnaire to assess social and emotional problems and competencies in 4 domains of behavior: behavioural dysregulation; externalising behavior problems; internalising behavior problems and competencies [87].
iv. Maternal measures
  1. Infant Observation [85] performed at baseline will be repeated at TEA, 12 months and 24 months corrected age. This will be performed by the Psychoanalytic Psychotherapist who is blinded to randomisation.
  2. Questionnaires performed at baseline will also be performed at TEA; Depression Anxiety Stress Scales [79], Edinburgh Postnatal Depression Scale [95] and Mother-to-infant Bonding Assessment [96]. Mothers will be asked to complete these assessments.
  3. At 12 months corrected age, maternal infant responsiveness and maternal postnatal attachment will be measured. The MIRI, a 22-item scale [82] will be performed. The MPAS which has 19 items, all scored on a 5-point scale, with 1 and 5 indicating low and high attachment respectively, will also be performed [101]. Mothers will be asked to complete this assessment.
  4. At 24 months corrected age, the Maternal Self Efficacy Scale (MSES) will be measured. The MSES is a 20-item measure of the mothers’ perceived self-competence of their maternal practice used at 12 months. The measure has good internal consistency (Cronbach’s **α** = 0.76-0.89) and is a good predictor of maternal competence, with strong concurrent validity with observation [102]. Mothers will be asked to complete this assessment.

### Healthy term born infants

Once written informed consent has been obtained, an appointment will be arranged for the infants to undertake the same assessments as the preterm cohort, at term equivalent age (39 to 42 weeks postmenstrual age) and infant observations at 12 months of age (Table 2).

**Table 2:** Outcome measures for term born infants

Measures for Term born infants	42 weeks post menstrual age	12 months corrected
Clinical	HNNE GMA NVS	
Infant Observation	IO	IO
Questionnaires	DASS MIBS EPDS	
Radiological	MRI	
Electrophysiological	dEEG	

HNNE = Hammersmith Neonatal Neurological Examination; GMA = General Movements Assessment; NVS = Neonatal Vision Scale; IO = Infant Observation; DASS = Depression, Anxiety, Stress Scale; MIBS = Mother to Infant Bonding Scale; EPDS = Edinburgh Postnatal Depression Scale; MRI = Magnetic Resonance Imaging; dEEG = dense array electroencephalography

### Blinding

Clinical assessors will be blinded to the intervention allocation. Likewise MRI and EEG data analyses will be performed in a blinded fashion.

### Potential confounders

Limitations include the single blinded nature of the study and the risk of potential contamination between intervention and CAU groups. Parents whose infants are allocated to the control group may be more likely to withdraw from the study after randomisation, if they perceive there is limited benefit to them.

### Data analysis

Summary statistics will be used to describe demographic and clinical data characteristics at baseline by allocated study treatment. Continuous data will be summarised using either mean and standard deviation, or median and inter-quartile range, depending on the distribution of the variable of interest. Categorical data will be presented as frequencies and percentages. Comparisons between the baseline values of the treatment groups will be conducted to assess the degree to which variables are comparable after randomisation. Between group differences will be investigated using linear regression for continuous data and Fisher’s exact test for categorical data. Potential confounding variables for the primary outcome are considered a-priori to be: gestational age at birth, birthweight, parity and maternal education. If any of these variables differ significantly between-groups (at *P* < 0.005), the identified variable(s) will be controlled for in the main analyses.

The primary study outcome is beta frequency global power measured in all VPT infants at term equivalent age. The mean difference between treatment groups will be calculated using linear regression with treatment group entered as the main effect. The corresponding 95 % Wald confidence interval and *p*-value will be reported. For secondary outcomes, we will present the effect estimate relating to a variable with a continuous outcome as a mean difference, which will be calculated using a linear regression model. If a continuous variable does not meet the assumptions necessary for linear regression, we will compare groups using the Mann–Whitney U Test. We will present the effect estimate relating to a variable with a binary outcome as an odds ratio, which will be calculated using a logistic regression model. We will present the effect estimate relating to a variable with a count outcome as an incident rate ratio, which will be calculated using a Poisson regression model. For all models the corresponding 95 % Wald confidence interval and p-value will be reported.

All analyses will primarily be undertaken using the ‘intention-to-treat’ approach, where all evaluable data is analysed in the treatment group according to which the participant was allocated, regardless of treatment received. Additionally, ‘per protocol’ analyses will be conducted where there have been large deviations from the planned intervention; all individuals with evaluable outcome data will be analysed according to the treatment they received (no massage/received < 50 % of total possible massage/received at least 50 % of total possible massage). Any such analyses will be clearly labeled as such and cautiously interpreted as perhaps indicating the maximum theoretical potential of the intervention. Statistical significance will be defined as alpha = 0.05. All tests conducted will be two-tailed.

## Discussion

This protocol paper outlines a randomised controlled trial of infant massage in VPT infants to detect effects on neurodevelopment. To our knowledge this study is the first to directly measure what influences infant massage may have on preterm brain structure and function using EEG, MRI and neurobehavioural assessments, and the mother-infant relationship in a preterm cohort.

## Abbreviations

AFD: Apparent fibre density
Bayley-III: Bayley Scales of Infant and Toddler Development, Third Edition
BDI: Beck Depression Inventory
BSID-II: Bayley Scales of Infant Development, Second Edition
DASS: Depression Anxiety Stress Scale
dEEG: Dense array electroencephalography
DWI: Diffusion weighted imaging
EEG: Electroencephalography
EPDS: Edinburgh Postnatal Depression Scale
FA: Fractional anisotropy
FOD: Fibre orientation distribution
GMA: General Movements Assessment
HARDI: High angular resolution diffusion imaging
HNNE: Hammersmith Neonatal Neurological Examination
HREC: Human research ethics committee
ITSEA: Infant Toddler Social and Emotional Assessment
MD: Mean diffusivity
MDI: Mental Developmental Index
MIBS: Mother-to-Infant Bonding Scale
MIRI: Maternal Infant Responsiveness Instrument
MPAS: Maternal Postnatal Attachment Scale
MRI: Magnetic Resonance Imaging
MSES: Maternal Self-Efficacy Scale
NVS: Neonatal Vision Scale
PDSS: Postpartum Depression Screening Scale
PMA: Postmenstrual age
PPD: Postpartum depression
SPSS: Statistical Package for the Social Sciences
TEA: Term equivalent age
TSE: Turbo spin echo
VPT: Very preterm infant
